# Complete Genome Sequence of *Xanthomonas arboricola* pv. juglandis 417, a Copper-Resistant Strain Isolated from *Juglans regia* L.

**DOI:** 10.1128/genomeA.01126-15

**Published:** 2015-10-01

**Authors:** Ulisses P. Pereira, Hossein Gouran, Rafael Nascimento, James E. Adaskaveg, Luiz Ricardo Goulart, Abhaya M. Dandekar

**Affiliations:** a Preventive Veterinary Medicine Department, State University of Londrina, Campus UEL, Londrina, PR, Brazil; b Plant Sciences Department, University of California Davis, Davis, California, USA; c Institute of Genetics and Biochemistry, Federal University of Uberlândia, Campus Umuarama, Uberlândia, MG, Brazil; d Department of Plant Pathology and Microbiology, University of California Riverside, California, USA

## Abstract

Here, we report the complete genome sequence of *Xanthomonas arboricola* pv. juglandis 417, a copper-resistant strain isolated from a blighted walnut fruit (*Juglans regia* L. cv. Chandler). The genome consists of a single chromosome (5,218 kb).

## GENOME ANNOUNCEMENT

The Gram-negative gammaproteobacterium *Xanthomonas arboricola* pv. juglandis, the causal agent of walnut blight, is the most important disease of Persian (English) walnut (*Juglans regia* L.) in California and many other production areas worldwide (1–3). The disease can occur on seedlings and mature walnut trees and is considered a major cause of yield reductions. The bacteria attacks leaves, buds, catkins, and young twigs, but fruits are generally most susceptible to infection. Symptoms begin as small dark-brown spots with a yellowish halo that develop into larger areas of dark, dead tissue. Most of the economic loss due to walnut blight is associated with fruit infection, which can lead to their premature drop and reduction in nut quality of fruit that remain on the tree (4–6). The disease is most severe in climates with rains throughout the growing season, however, in California the disease develops during spring rains. Host susceptibility differs among cultivars (7); however, all commercially grown walnut varieties are considered susceptible to walnut blight (8).

The current management strategy for walnut blight is mostly based on multiple applications of copper-based bactericides for protecting susceptible plant tissue, but efficacy is often variable (9). Extensive copper usage for the past decades has caused selection for copper-resistant strains (10, 11). We describe here the genome sequence of *X. arboricola* pv. juglandis strain 417, a copper-resistant strain isolated from a blighted cv. Chandler walnut fruit in Chico, California, in 2012.

The genome sequence was obtained using the MiSeq (Illumina Inc., San Diego, CA) system with two paired-end libraries, which generated 10,403,000 and 9,233,152 (both with reads of 100 bp in size) and insert size of 500 bp and 2,000 bp, respectively. The estimated genome coverage with these two libraries was ~385-fold. After sequencing, reads were assembled using Mira 4.0 software (12) resulting in 59 contigs, *N*_50_ of 171,731 bp, and the smallest contig of 1,102 bp. These contigs were ordered using the Contiguator software (13) against many genomes of the same genus. The genome of *X. campestris* strain 17 (GenBank accession number CP011256) was used as a reference due to better synteny and number of contigs mapped. The initial scaffold was later subjected to a finishing process using CLC Genomics Workbench software, and gaps were removed with recursive rounds of short reads mapped against the scaffold (14). The annotation step was performed using NCBI Prokaryotic Genome Annotation Pipeline.

The final genome had five large contigs separated by four gaps, the total size was 5,218,943 nucleotides with 4,178 putative open reading frames. The G+C content was 65.41%, there were three rRNA genes, 52 tRNA genes, and 133 predicted pseudogenes. Further analysis of the genome is now under way. It will allow us to identify specific factors that might explain the differences in the pathobiology of *X. arboricola* pv. juglandis when compared to other members of the *Xanthomonadaceae* family.

### Nucleotide sequence accession number.

The *Xanthomonas arboricola* pv. juglandis strain 417 genome sequence and annotation data have been deposited at DDBJ/EMBL/GenBank under the accession number CP012251.
